# MRI-based evaluation of multiorgan iron overload is a predictor of adverse outcomes in pediatric patients undergoing allogeneic hematopoietic stem cell transplantation

**DOI:** 10.18632/oncotarget.19021

**Published:** 2017-07-05

**Authors:** Natalia Maximova, Massimo Gregori, Giulia Boz, Roberto Simeone, Davide Zanon, Giulia Schillani, Floriana Zennaro

**Affiliations:** ^1^ Bone Marrow Transplant Unit, Institute for Maternal and Child Health–IRCCS Burlo Garofolo, 34137 Trieste, Italy; ^2^ Department of Radiology, Institute for Maternal and Child Health–IRCCS Burlo Garofolo, 34137 Trieste, Italy; ^3^ University of Trieste, Piazzale Europa, 34128 Trieste, Italy; ^4^ Department of Transfusion Medicine, Institute for Maternal and Child Health–IRCCS Burlo Garofolo, 34137 Trieste, Italy; ^5^ Pharmacy, Institute for Maternal and Child Health–IRCCS Burlo Garofolo, 34137 Trieste, Italy

**Keywords:** multiorgan iron overload, pediatric patients, allogeneic hematopoietic stem cell transplantation, transplant-related complications

## Abstract

The medical records of 44 pediatric patients who underwent allogeneic transplantation from 2011 to 2015 were retrospectively reviewed. Magnetic resonance imaging was used to measure iron concentrations in the liver, spleen, pancreas and bone. These patients were divided into two groups, 18 with non-elevated (< 100 μmol/g; Group 1) liver iron concentration before transplantation and 26 with elevated (> 100 μmol/g; Group 2) concentration . We compared transplant-related outcomes in the two groups. Iron overload was a negative prognostic risk factor for sinusoidal obstruction syndrome (OR = 17), osteoporosis (OR = 6.8), pancreatic insufficiency (OR = 17) and metabolic syndrome (OR = 15.1). No statistically significant differences in overall survival, disease-free survival, relapse incidence and incidence of acute or chronic graft-versus host disease were observed between the two groups. Mean times to engraftment of platelets (43.0 ± 35.3 days vs. 22.1 ± 9.5 days, *p* < 0.05) and neutrophils (23.1 ± 10.4 days vs. 17.8 ± 4.6 days, *p* < 0.05) appear significantly longer in Group 2 than in Group 1. Time to platelet engraftment showed statistically significant correlation with pre-transplant liver (*r* = 0.5775; *p* < 0.001) and bone iron concentration (*r* = 0.7305; *p* < 0.001). Post-transplant evaluation pointed out that iron concentration analyzed at the first follow-up peaked in all tissues. The iron accumulation was highest in bone, followed by the spleen, liver and pancreas. One year post transplant 9 of 18 (50%) patients in Group 1 and 6 of 22 (27%) in Group 2 presented with bone and/or spleen iron overload, but not with liver overload. Liver iron concentration is not always a reliable indicator of systemic siderosis or of the efficacy of chelation therapy.

## INTRODUCTION

Iron overload has been associated with poor prognosis in patients undergoing allogeneic hematopoietic stem cell transplantation (HSCT) for hematological malignancies and myelodysplastic syndrome (MDS) [1–3]. Iron overload was shown to correlate with an increased risk of non-relapse mortality following HSCT and may enhance the risks of acute and chronic graft versus host disease (GVHD) [1–3]. Transfusion iron overload increases the risk of infection, sinusoidal obstruction syndrome (SOS) and hepatic dysfunction during the post-transplant period. Because humans lack a physiologic mechanism for iron excretion, excess iron could persist for years after HSCT. Additionally, iron homeostasis is frequently complicated by factors that increase hepcidin expression, including inadequate erythropoiesis, inflammation, infections and hypoxia [4]. Iron toxicity is due largely by redox-active, non-transferrin-bound iron, which can freely cross membrane barriers [5, 6]. Clinical evidence indicates that iron-associated toxic effects are likely when liver iron concentration (LIC) exceeds a threshold of 90–125 µmol/g (5–7 mg/g) dry weight, concentrations that have been associated with liver fibrosis as well as with cardiac and pancreatic insufficiency [7].

The ability of magnetic resonance imaging (MRI) to non-invasively measure tissue iron concentration in humans and the development of new iron-chelating agents have resulted in a dramatic improvement in the survival of patients with severe iron overload [8–10].

Although hepatic and cardiac iron overload have been extensively studied, limited data are available on iron overload in other organs. Few studies have assessed the correlations among degrees of siderosis in different tissues of patients with thalassemia, and relatively little is known about associations between multi-organ siderosis and immediate and long-term effects in pediatric HSCT recipients [11–17].

The use of MRI for liver iron evaluation is an established method to assess iron content, this is not so effective for bone, pancreas and spleen.

Our Institute routinely uses MRI with various gradient-recalled-echo (GRE) sequences to quantitatively measure LIC in all pediatric patients before and after allogeneic HSCT [18]. MRI findings in these patients were reviewed, and MRI-determined quantitation of iron concentrations in other organs, including the spleen (SIC), pancreas (PIC) and bones (BIC), were analyzed.

## RESULTS

### Relationships among pre-transplant LIC, transfusion history and post-transplant tissue iron concentration

During the study period, 44 pediatric patients underwent HSCT; their baseline characteristics are shown in Table 1. The presence or absence of iron overload was based on pre-transplant MRI-determined LIC, which has been shown to be as reliable as liver biopsy [19]. Moreover, multiple regression analysis showed significant correlations of pre-transplant LIC, PIC, SIC, BIC and baseline ferritin levels (LIC maximum R-square corrected 0.52098; *p <* 0.05).

**Table 1:** Baseline characteristics of patients of the study group

Baseline characteristics	Value	Group 1		Group 2	**p Value
		LIC < 100 µmol/g		LIC > 100 µmol/g	
**Patients (%)**	44 (100)	18 (40.9)		26 (59.1)	
**Gender (%)**					
Male	27 (61.4)	12 (44.4)		15 (55.6)	NS
Female	17 (38.6)	6 (35.3)		11 (64.7)	NS
**Age, years, median at transplant (range)**	8.5 (0–17)	8.3 (0–17)		8.3 (0–16)	NS^†^
**Underlying disease (%)**					
ALL	21 (47.7)	8 (38.1)		13 (61.9)	NS
AML	8 (18.2)	0		8 (100)	< 0.05
MDS	5 (11.4)	2 (40.0)		3 (60.0)	NS
Hemoglobinopathy	2 (4.5)	1 (50.0)		1 (50.0)	NS
Inherited disease^#^	7 (15.9)	6 (85.7)		1 (14.3)	NS
Solid tumor	1 (2.3)	1 (100)		0	NS
Disease stage* (%)					
Early	8 (23.5)	2 (25.0)		6 (75.0)	NS
Intermediate	11 (32.4)	8 (63.6)		3 (36.4)	< 0.05
Late	15 (44.1)	0		15 (100)	< 0.05
**Mean time between onset and transplant, months (%)**					
< 12	17 (38.6)	6 (35.3)		11 (64.7)	NS
> 12	27 (61.4)	12 (44.4)		15 (55.6)	NS
**Mean number of PRBC units received (range)**					
Before transplant	21.4 (0–78)	9.8 (0–51)		30.0 (7–78)	< 0.001^†^
After transplant	11.7 (0–67)	5.1 (0–18)		16.6 (0–67)	< 0.001^†^
**Mean ferritin (ng/mL) pre–transplant (range)**					
	2389.8 (89–29961)	655.4 (89–2358)	3416.4 (546–2996)		< 0.05^†^
**Donor type (%)**					
Matched related donor	16 (36.4)	9 (56.2)	7 (43.8)		NS
Matched unrelated donor	20 (45.4)	8 (40.0)	12 (60.0)		NS
Haploidentical donor	5 (11.4)	1 (20.0)	4 (80.0)		NS
**Graft source (%)**					
Bone marrow	39 (88.6)	18 (46.2)^§^	21 (53.8)		NS
PBSC	2 (4.5)	0	2 (100)		NS
Umbilical cord blood	3 (6.8)	0	3 (100)		NS
**Conditioning (%)**					
Myeloablative:	40 (90.9)	14 (35.0)	26 (65.0)		< 0.05
TBI – based	16 (40.0)	5 (31.3)	11 (68.7)		NS
BU – based	24 (60.0)	9 (37.5)	15 (62.5)		NS
Reduced intensity	4 (9.1)	4 (100)	0		< 0.05
**Karnofsky/Lansky performance score at transplant (%)**					
> 80%	39 (88.6)	18 (46.2)^§^	21 (53.8)		NS
< 80%	5(11.4)	0	5(100)		NS
**Gilbert syndrome (%)**	12 (27.3)	3 (25.0)	9 (75.0)		NS

*Disease stage was defined according to previously published classification. This classification is applied to patients with acute leukemia and MDS only [45].

**Fisher’s test.

^†^ Mann Whitney’s test.

^#^ Primary immunodeficiency, osteopetrosis, inborn error of metabolism.

^§^All patients of Group 1.

NS: not significant

LIC: liver iron concentration; sd: standard deviation; ALL: acute lymphoblastic leukemia;

AML: acute myeloid leukemia; MDS: myelodysplastic syndrome; PRBC unit: packed red blood cell unit;

TBI: total body irradiation; BU: busulfan.

Of the 44 patients, 18 had pre-transplant LIC < 100 µmol/g, indicating normal iron concentration or a mild iron overload (Group 1), and 26 had pre-transplant LIC > 100 µmol/g, indicating moderate to severe iron overload (Group 2). Mean pre-transplant LICs in these two groups were 53.3 ± 18.9 µmol/g and 201.5 ± 67.4 µmol/g, respectively. Most patients in Group 1 presented with early stage acute lymphocytic leukemia (ALL) or an inherited disease, whereas most patients in Group 2 presented with late stage ALL or acute myelogenous leukemia (AML). All patients in Group 2 had a Karnofsky/Lansky score < 80%, and most (75%) were affected by Gilbert syndrome.

As expected, pre-transplant iron overload in these patients correlated with ferritin concentration and the number of packed red blood cell (PRBC) units transfused before HSCT. The mean baseline ferritin concentration was five times as high (*p <* 0.05), and the number of PRBC units transfused per patient was approximately three times as high (29.3 ± 17.4 vs. 9.9 ± 11.5, *p <* 0.001), in Group 2 than in Group 1 (Table 1).

Comparisons of PIC (47.7 ± 62.4 µmol/g vs. 11.7 µmol/g, *p <* 0.05), SIC (219.4 ± 85.2 µmol/g vs. 64.2 ±65.3 µmol/g, *p <* 0.001) and BIC (225.8 ± 59.0 µmol/g vs. 145.3 ± 74.7 µmol/g, *p <* 0.001) showed that all of these concentrations were significantly higher in Group 2 than in Group 1. Mean PIC values were lower than mean SIC and BIC values in both groups. Of the 18 patients in Group 1, only two had normal iron concentrations in all organs. Three patients had mild iron overload localized in a single organ, two with hepatic and one with splenic iron overload; three patients presented with mild iron overload involving the liver and one additional organ, with two having LICs lower than SIC or BIC. The remaining 10 patients presented with mild iron overload involving the liver and two other organs, with nine of these patients having a lower grade of liver siderosis compared to the other involved organs. None of these patients in Group 1 presented with iron overload in all four organs analyzed.

**Figure 1:**
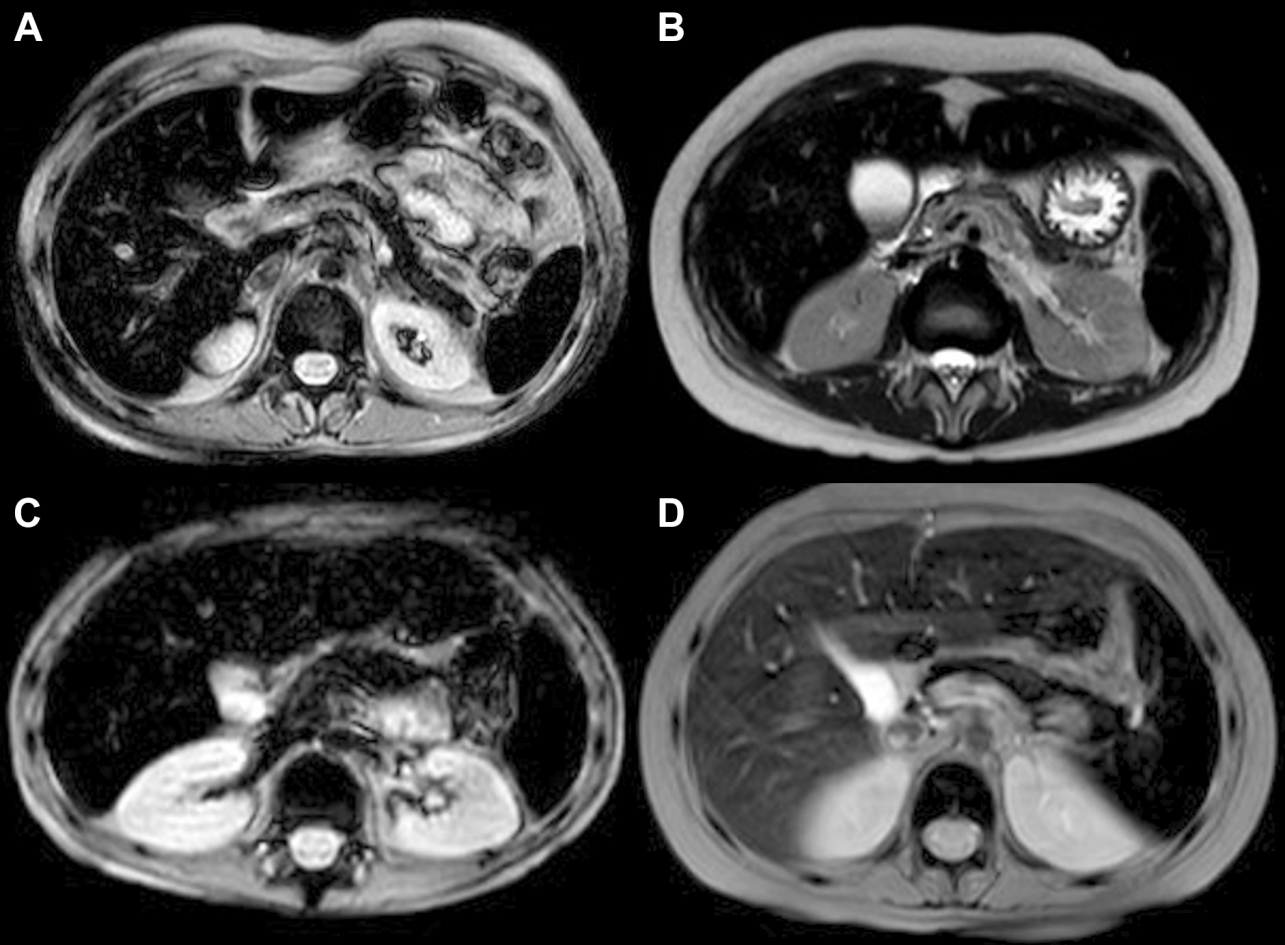
Abdominal MRI T2 FFE (fast field echo) sequences show hypointense liver, spleen, pancreas and bone signal due to abnormal iron deposition (**A**, **B**) patients with intense transfusion regimen history; (**C**) patient with only 5 transfusion before MRI quantification of iron concentration; (**D**) patient treated with deferasirox: minor liver hyperintensity compared to spleen, pancreas and bone intensity.

None of the 26 patients of the Group 2 presented with pathological iron concentrations in one or two organs: 20 patients presented with iron overload in three organs and six in all four organs analyzed (Figure 1A, 1B).

Table 2 shows correlations of pre-transplant multi-organ iron concentrations with the number of PRBC units transfused before HSCT and pre-transplant ferritin concentrations. Although LIC, SIC and BIC correlated significantly with the number of PRBC units transfused, there was no correlation between PIC and the number of PRBC units.

**Table 2:** Correlation between different tissues iron concentration and transplant outcome variables

Transplant variables	LIC	BIC	SIC	PIC
	µmol/g	µmol/g	µmol/g	µmol/g
**Number of PRBC units received**	*r* = 0,6785	*r* = 0,5288	*r* = 0,7368	*r* = 0,2183
	*p <* 0,001	*p <* 0,001	*p <* 0,001	NS
**Ferritin pre-transplant (ng/mL)**	*r* = 0,7371	*r* = 0,5197	*r* = 0,7365	*r* = 0,3249
	*p <* 0,001	*p <* 0,001	*p <* 0,001	*p <* 0,05
**Neutrophil engraftment (days)**	*r* = 0,4233	*r* = 0,4762	*r* = 0,2567	*r* = 0,3650
	*p <* 0,001	*p <* 0,001	*p <* 0,05	*p <* 0,001
**Platelet engraftment (days)**	*r* = 0,5775	*r* = 0,7305	*r* = 0,4656	*r* = 0,4461
	*p <* 0,001	*p <* 0,001	*p <* 0,001	*p <* 0,001

LIC: liver iron concentration; BIC: bone iron concentration; SIC: spleen iron concentration;

PIC: pancreas iron concentration; PRBC unit: packed red blood cell unit.

Pre-transplant PIC, SIC and BIC of each patient were compared with post-transplant MRI-determined iron accumulation 1–6, 6–12 and 12–24 months. Comparisons of baseline data with results of the first follow-up MRI at time F1 (1–6 months), performed after complete engraftment and the end of transfusion dependence, showed a general worsening of iron accumulation. Of the 18 patients in Group 1, none showed complete absence of iron overload or siderosis in a single organ, four presented with siderosis of two organs, including two with iron overload in the spleen and bone but not in the liver; and 14 presented with siderosis of the liver and other two organs. Of these 14 patients, 12 had LIC values lower than SIC and BIC values. None of the patients in Group 1 presented with siderosis of all four organs, because none presented with siderosis of the pancreas.

Of the 26 patients in Group 2, none showed a total absence of iron overload or siderosis involving fewer than three organs. Among the 20 patients who presented pre-transplant siderosis involving three organs, nine showed onset of pancreatic iron accumulation, increasing the number of patients with siderosis in all four organs from 6 to 15.

The percentage of patients with a higher grade of hepatic siderosis at F1 than at baseline was significantly higher in Group 2 than in Group 1 (29.6% vs 11.8%), whereas the percentage of patients with major number of organs with siderosis respect basilar data was similar in Groups 1 and 2 (41.2% vs. 37.0%).

The mean number of PRBC units received by patients in both groups was lower in post-transplant period compared to the period from diagnosis to admission in the Transplant Center. However, the mean number of post-transplant PRBC units received by patients was significantly higher in Group 2 than in Group 1 (16.6 vs. 5.1 units, *p* < 0.05; Table 1).

**Figure 2:**
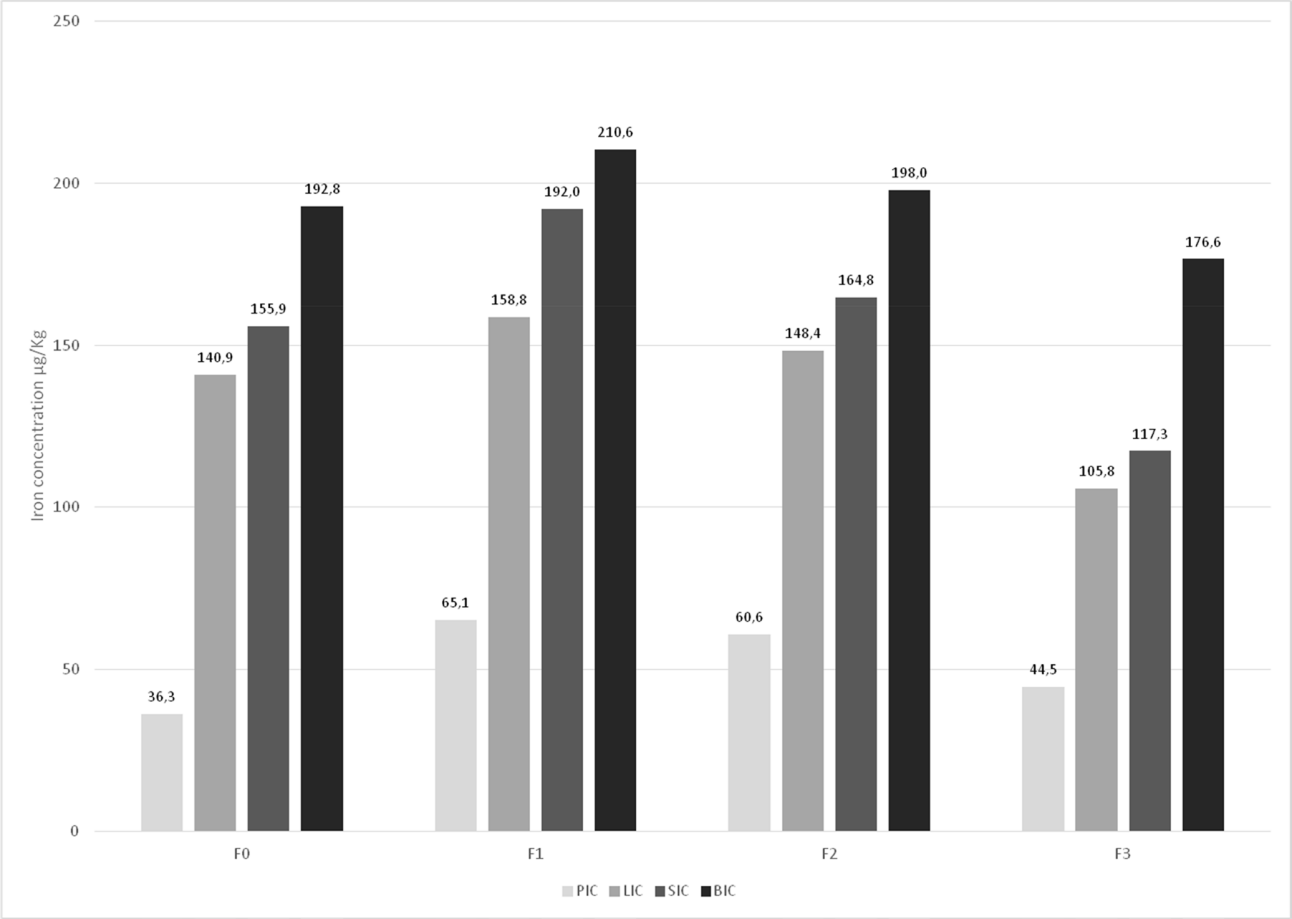
Pancreatic iron concentration (PIC), liver iron concentration (LIC), spleen iron concentration (SIC) and bone iron concentration (BIC) at the pre-transplant (F0) and post-transplant (F1, F2 and F3) MRI-based evaluation All patients in the study (*n* = 44) are being shown here. Multiple comparison: Friedman test: PIC: *p <* 0.05; LIC: *p <* 0.05; SIC: *p <* 0.05; BIC: *p >* 0.05 (NS). Wilcoxon test: PIC: F0 vs F1: *p <* 0.05 – LIC: F0 vs F1: *p <* 0.05; F0 vs F2: *p <* 0.05 – F0 vs F2: *p >* 0.05 (NS); F0 vs F3: *p >* 0.05 (NS) – F0 vs F3: *p <* 0.05; F1 vs F2: *p >* 0.05 (NS); F1 vs F2: *p >* 0.05 (NS); F1 vs F3: *p <* 0.05 – F1 vs F3: *p <* 0.05 – F2 vs F3: *p <* 0.05 – F2 vs F3: *p <* 0.05; SIC: F0 vs F1: *p <* 0.05 – BIC: F0 vs F1: *p <* 0.05; F0 vs F2: *p >* 0.05 (NS); F0 vs F2: *p >* 0.05 (NS) – F0 vs F3: *p <* 0.05; F0 vs F3: *p >* 0.05 (NS) – F1 vs F2: *p <* 0.05; F1 vs F2: *p >* 0.05 (NS) – F1 vs F3: *p <* 0.05; F1 vs F3: *p <* 0.05; F2 vs F3: *p <* 0.05; F2 vs F3: *p <* 0.05.

Post-transplant MRI evaluation of iron overload showed that iron concentration peaked at F1 in all tissues analyzed. Iron accumulation was highest in bone, followed by the spleen, liver and pancreas. Iron accumulation tended to decrease at the F2 (6–12 months) and F3 (12–24 months) follow-up evaluations (Figure 2).

### Relationship between pre-transplant liver iron concentration and post-transplant outcomes

Iron overload was found to be a negative prognostic risk factor for the development of important clinical complications, including SOS (OR = 17), osteoporosis (OR = 6.8), pancreatic insufficiency (OR = 17) and metabolic syndrome (OR = 15.1). Elevated pre-transplant LIC values showed a high sensitivity, but a lower specificity, in predicting the development of all four adverse outcomes (Table 3). In contrast, elevated LIC was not associated with either GVHD or relapse. Statistically significant increases in LIC were associated with a higher rate of infections (*p <* 0.05), but pre-transplant LIC > 100 μmol/g was not significantly associated with the development of infections (OR = 2.1, *p >* 0.05). Statistically significant increases in LIC were associated with transplant-related mortality (TRM), although higher pre-transplant LIC was not (OR = 7.4, *p >* 0.05), a result that may have been due to the small number of patients (*n =* 44) and few deaths (*n =* 5).

**Table 3:** Correlation between pre-transplant LIC and transplant outcome variables

	Outcome	Frequency		Lic		Sensitivity	Specifity	Odds ratio		
		***n*°**	**%**	**µmol/g**	P*	**%**	**%**	**OR**	**95% confidence interval**	***P***
**SOS/liver injury**	negative	30	68.2	114 ± 88	< 0,05	93	57	17,0	1,963–147,2	< 0,05
	positive	14	31.8	199 ± 68						
**Osteoporosis**^#^	negative	26	59.1	110 ± 81	< 0,05	83	58	6,8	1,577–29,47	< 0,05
	positive	18	40.9	185 ± 87						
**Metabolic syndrome**^ϰ^	negative	25	56,8	99 ± 71	< 0,05	89	64	15,1	2,83–80,9	< 0,05
	positive	19	43,2	196 ± 86						
**Pancreatic insufficiency**^†^	negative	35	79,5	113 ± 73	< 0,05	100	50	17	0,91–316,7	< 0,05
	positive	9	20,5	249 ± 73						
**GVHD acute/chronic**	negative	23	52,3	140 ± 107	NS	71	52	2,7	0,78–9,53	NS
	positive	21	47,7	142 ± 70						
**Infections**	negative	11	25	94 ± 72	< 0,05	63	54	2,1	0,52–8,37	NS
	positive	33	75	157 ± 91						
**Relapse incidence**	negative	35	79,5	138 ± 92	NS	55	40	0,8	0,19–3,66	NS
	positive	9	20,5	151 ± 91						
**Transplant related**	negative	39	88,6	133 ± 84	NS	100	45	7,4	0,37–146,6	NS
**mortality**	positive	5	11,4	206 ± 122						

*Mann-Whitney test.

^#^Dual-energy X-ray absorptiometry (DXA) was used to measure bone mineral density.

^ϰ^Metabolic syndrome was defined according WHO criteria [46].

Pathological values of serum amylase, lipase and fecal elastase-1 (exocrine pancreatic function); insuline, c-peptide, hemoglobin A1c (endocrine pancreatic function).

LIC: liver iron concentration; SOS: sinusoidal obstruction syndrome; GVHD: graft versus host disease.

### Relationship between multi-organ iron overload and post-transplant outcome

**Figure 3:**
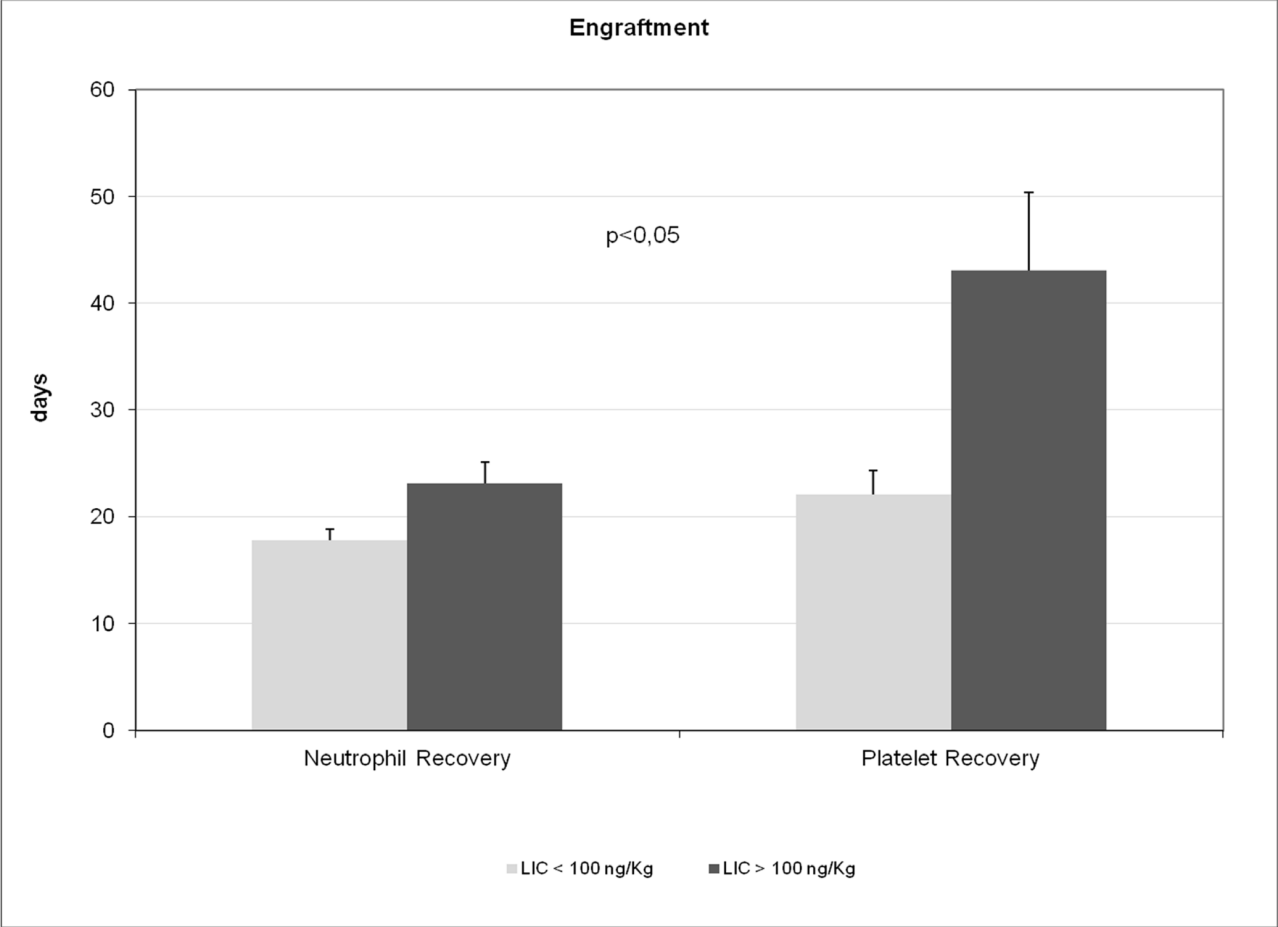
Time to engraftment of platelets and neutrophils in Group 1 (pre-transplant LIC < 100 µmol/g) vs Group 2 (pre-transplant LIC > 100 µmol/g) Note: value are mean ± standard error of mean.

To assess the prognostic effects of multi-organ siderosis, this study focused on transplant outcomes, such as engraftment, overall survival, TRM, disease recurrence, and quality of life, as primary endpoints. Pre-transplant LIC was significantly associated with neutrophil and platelet engraftment, with mean times to engraftment of both platelets (43.0 ± 35.3 days vs. 22.1 ± 9.5 days, *p <* 0.05) and neutrophils (23.1 ± 10.4 days vs. 17.8 ± 4.6 days, *p <* 0.05) being significantly longer in Group 2 than in Group 1 (Table 4, Figure 3). Time to platelet engraftment showed statistically significant correlations with pre-transplant LIC (*r* = 0.5775; *p <* 0.001) and BIC (*r* = 0.7305; *p <* 0.001). Pre-transplant LIC, PIC, SIC, and BIC showed statistically significant correlations with mean times to platelet and neutrophil engraftment (Table 2).

**Table 4:** Impact of pre-HSCT liver iron concentration on transplant outcome variables and iron concentration in other tissue

Outcome variables	Group 1	Group 2	*P*-value
	LIC < 100 µmol/g	LIC > 100 µmol/g	
**Engraftment, days (± sd)**			
Neutrophil recovery (> 0.5 × 10^9^/l)	17.8 ± 4.6	23.1 ± 10.4	< 0.05
Platelet recovery (> 20 × 10^9^/l)	22.1 ± 9.5	43.0 ± 35.3	< 0.05
**Transplant related mortality (%)**^†^			
At day + 360	0	5 (19.2)	NS**
**Relapse incidence (%)**^#^	4 (22.2)	5 (19.2)	NS**
**Relapse mortality**	1 (5.5)	2 (7.7)	NS**
Disease-free survival (%)*	14 (77.8)	17 (65.4)	NS**
**Overall survival (%)^**	17 (94.4)	19 (73.1)	NS**
**Karnofsky/Lansky score at day +360 (%)**			
80–100 %	16 (94.1)	16 (84.2)	NS**
< 80 %	1 (5.9)	3 (15.8)	
**Early (< 6 months) complications (%)**			
Infections	11 (61.1)	15 (57.7)	NS**
Fungal pneumonia	0	6 (23.1)	NS**
Bacterial pneumonia	2 (11.1)	3 (11.5)	NS**
SOS/ hepatic injury	1 (5.6)	13 (50.0)	< 0.05
Acute GVHD^¶^	6 (33.3)	13 (50.0)	NS**
Metabolic syndrome	2 (11.1)	17 (65.4)	< 0.05
**Late (> 6 months) complications (%)**			
Chronic GVHD^¶^	0	2 (7.7)	NS**
Pancreatic exocrine insufficiency^ϰ^	0	9 (34.6)	< 0.05
Osteoporosis	3 (16.7)	15 (57.7)	< 0.05
Chronic infections	1 (5.6)	6 (23.1)	NS**
Secondary graft failure	1 (5.6)	7 (26.9)	NS**

^†^Transplant related mortality was defined as death without relapse/disease progression.

^#^Relapse incidence was defined as time to onset of leukemia recurrence.

*Disease-free survival was defined as survival without relapse/disease progression.

^^^Overall survival was defined as the time to death from any cause.

^¶^GVHD grade II-IV of any organ.

^ϰ^8 of 9 patients with pancreatic exocrine insufficiency have had from moderate to severe pancreatic iron overload.

HSCT: hematopoietic stem cell transplantation; LIC: liver iron concentration; PIC: pancreas iron

concentration; SIC: spleen iron concentration; BIC: bone iron concentration; sd: standard deviation;

SOS: sinusoidal obstruction syndrome; GVHD: graft versus host disease.

Of the 44 patients, five (11.4%) died of transplant-related causes after 12 months. Increased pre-transplant LIC was significantly associated with TRM, as all five patients who died of a cause unrelated to the primary disease belonged to Group 2. Two patients died due to a fungal infection, one patient died due to a viral infection, one patient due to SOS and one after a GVHD. No statistically significant differences in overall survival, disease-free survival and relapse incidence were observed between the two groups (Table 4).

The effects of multi-organ iron overload on the development of early and late transplant complications were also evaluated. The percentages of patients with SOS/hepatic injury (50.0% vs. 5.6%) and metabolic syndrome (65.4% vs. 11.1%) were significantly higher in Group 2 than in Group 1. Assessments of late complications showed that the percentages of patients with osteoporosis and secondary graft failure were higher in Group 2 (*p <* 0.05). Moreover, all patients with pancreatic insufficiency belonged to Group 2. The percentages of patients with acute and chronic GVHD, however, did not differ significantly in the two groups (Table 4).

## DISCUSSION

The primary aim of this study was to determine whether evaluation and monitoring of iron loading in organs other than the liver could better predict transplant outcomes than routine measurements of LIC. This study, which involved a relatively small number of pediatric patients who underwent allogeneic HSCT at a single medical center, found that most of these patients presented with severe but heterogeneous abdominal parenchymal iron overload after transplantation. The heterogeneous distribution of iron loading among different organs was expected, because abdominal organs have different mechanisms and rates of iron uptake [20]. However, the high percentage of patients presenting with severe siderosis at the time of transplantation was unexpected.

This study showed that iron overload in patients with hematological malignancies is multifactorial, and not due exclusively to the intense transfusion regimen. Previously, systemic iron overload was studied only in transfusion-dependent patients with beta-thalassemia and sickle cell anemia. Iron overload in these patients was found to occur after transfusion of about 20 PRBC units [21], with iron deposited in parenchymal tissues after approximately 1 year of transfusions [22]. In contrast, our study found that nine of the 26 patients in Group 2 presenting with pre-transplant iron overload had received fewer than 20 PRBC units, with two receiving fewer than 10 units (Figure 1C). Furthermore, the time interval between the first transfusion and the appearance of multi-organ iron deposition in 15 of these 26 patients was shorter than 1 year. These findings suggest that patients with hematological malignancies develop iron overload faster, and after receiving fewer PRBC units, than patients with beta-thalassemia and sickle cell anemia.

In thalassemic patients, siderosis is caused mainly by the oversupply of exogenous iron from transfused PRBCs, whereas, in patients with malignant disorders, a substantial fraction of iron overload is not due to transfused blood. Intensive cytotoxic therapy before HSCT destroys both bone marrow and neoplastic cells, releasing internal iron contents from cells and consequently increasing free iron concentrations [23]. Furthermore, high dose chemotherapy or total body irradiation, as part of conditioning prior to HSCT, can damage hepatic cells, which release cellular iron pools and contribute to further increases in iron load [24]. This hypothesis is confirmed by the finding that most patients in this study with severe iron overload had late stage leukemia, either AML or ALL, and that patients with AML had received short, but highly intense, cytotoxic regimens and that patients with ALL had received many long chemotherapy cycles.

A second important result of this study was our finding that LIC is not a reliable indicator of total body iron stores or of the efficacy of chelation therapy. This finding is in contrast with previous results, in that MRI-based LIC is universally considered an excellent indicator of systemic iron overload, because of its optimal correlation with the liver biopsy.

Of our 18 patients without pre-transplant hepatic iron load (Group 1), 13 (72.2%) presented with moderate, or even severe, iron overload in bone and/or spleen 1–6 months later. Moreover, of the 26 patients with baseline iron overload (Group 2), 10 (38.5%) presented with a higher grade iron overload in one or more organs than in liver. At the three post-transplant follow-ups, the same percentages of patients in Group 2 presented with BIC and/or SIC higher than LIC. Over time, iron concentrations decreased in all abdominal organs, with LIC showing the most significant reduction.

One year after transplant, at F3, nine of 18 (50%) patients in Group 1 and six of 22 (27%) in Group 2 presented with bone and/or spleen iron overload, but their LICs were normal.

We have not observed a significant reduction of late complications with the reduction of iron overload, but the number of patients with this kind of events was really small.

The finding, that the percentage of patients presenting with pathological iron content at F3 was higher in Group 1 than in Group 2, was unexpected. It was likely due to the administration of chelation therapy only to patients in Group 2, not to those in Group 1. For outpatients the chelation therapy consisted of the administration of deferasirox, which more effectively removes iron from the liver than from other organs [25], resulting in a more rapid normalization of LIC than of BIC and SIC (Figure 1D). These results underscore the importance of organ-specificity of chelation therapy: “More importantly, dangerous heart iron accumulation and cardiac dysfunction can occur despite apparently superb control of liver iron during prospective longitudinal evaluation” [26].

This study confirms the correlations between iron overload and the risks of early and late transplant-related complications, such as SOS, infections, pancreatic insufficiency, and metabolic syndrome, in transplant recipients with systemic siderosis. In addition, these results confirm the lack of correlation between iron overload and the rates of acute and chronic GVHD and of relapse [27].

Another important finding of this study was the close correlations between pre-transplant BIC and times to neutrophil and platelet engraftment (*p <* 0.001 each). MRI-based assessments showed that BIC was higher than LIC, SIC, and PIC at all four time points, both before (F0) and after (F1–F3) transplantation. A significant amelioration of hematopoiesis has been associated with decreased transfusion requirements in patients with MDS and aplastic anemia [28, 29]. Moreover, iron overload was recently supported to significantly delay hematopoietic recovery after bone marrow transplantation, indicating that iron accumulation in the bone affects the hematopoietic microenvironment [30]. Bone marrow is a critical microenvironment, regulating many activities of stem cells, including self-renewal, mobilization, engraftment, and lineage differentiation [31].

Our results also showed that, in contrast to the correlations between the number of transfused PRBC units and iron overload in the liver, spleen and bone, there was no correlation between PRBC units trasfused and pancreatic iron overload. The kinetics of iron uptake and clearance differ in the liver and in extrahepatic tissues, such as the pancreas, in that the latter almost selectively load circulating non–transferrin-bound iron (NTBI) [32]. Pancreatic iron overload is quite frequent among patients with transfusion-dependent anemias [33], but is uncommon among patients with hematologic malignancies. Of our 44 patients, eight (18.2%) developed pancreatic iron overload after transplantation, with all eight having baseline severe hepatic iron overload (Group 2). At baseline, three of these patients presented with moderate pancreatic iron overload (PIC = 100–200 µmol/g), whereas five had normal PIC (< 36 µmol/g). Due to their severe pre-transplant liver iron overload, all eight of these patients were evaluated by MRI 1 month after transplantation. The three patients with increased PIC at baseline showed worsening of siderosis grade and developed severe pancreatic iron overload (PIC >200 µmol/g). The five patients with normal pre-transplant PIC developed moderate to severe pancreatic iron overload (PIC = 170–230 µmol/g). The mean number of PRBC units transfused into each patient in the interval between pre-transplant and first post-transplant MRI evaluations was 5.25 (range 4–8).

Irradiation and chemotherapy-related damage may accelerate iron deposition in pancreatic tissue, by a mechanism similar to that of anthracycline treatment of cardiac myocytes [34–38].

Clinical implications of the spleen iron overload are little-known. Splenomegaly due to iron saturation is the most common and also the most investigated side effect related to spleen iron overload. Recurrent infections have been reported in patients with a variety of iron-overload disorders. From *in vitro* studies iron and iron-binding proteins have been shown to impair a variety of immunological functions [39, 40] and also to interfere with non-specific defence mechanisms [41]. Good et al., have shown that iron overload was associated with a reduction in the ability of spleen cells to mount an allogeneic cytotoxic response [42].

Interestingly, nine of the 12 (75%) patients in our study with Gilbert syndrome showed severe pre-transplant siderosis and were therefore included in Group 2. A reduction in activity of the enzyme uridine diphosphate–glucuronyl transferase may affect the metabolism of drugs and may represent a pharmacogenetic risk factor for drug toxicity [43]. Reduced enzyme activity may also promote tissue iron deposition.

In conclusion, pre-transplant assessment of systemic iron overload based on iron concentration in four organs allows to estimate as precisely as possible the total body iron pool and to monitor the effectiveness of chelation treatment. Early introduction of chelation therapy can significantly improve transplant-related outcomes. This treatment may be especially useful in preventing the pre-transplant onset or worsening of siderosis in patients subjected to chemotherapy of treatment intensity level 3 or 4 [44].

## MATERIALS AND METHODS

### Study design and data collection

The study protocol was approved by the Ethics Committee of the Institute for Research in Maternal and Child Health Burlo Garofolo of Trieste, Italy (reference no. 1105/2015), which waived the requirement for informed consent because of the retrospective nature of this study. Written informed consent for the use of any clinical data in research was obtained from all patients and their parents or guardians at the time of admission to the Transplant Unit.

The medical records of 44 consecutive pediatric patients who underwent allogeneic HSCT in our Institute from March 2011 to March 2015 were retrospectively reviewed and analyzed. Patients were included if they were <18 years old at the time of HSCT, had been followed-up for more than 1 year, and had undergone MRI quantification of LIC at our Institute before transplantation and at least three times after transplantation, with all MRI results available for investigation.

To assess tissue iron content we calculated the liver, spleen, pancreas and bone to muscle ratio for every sequence. Iron concentration values < 40 µmol/g were classified as normal, iron concentration values of 40–100 µmol/g were classified as mild iron overload, values of 100–200 µmol/g as moderate iron overload and iron concentration values > 200 µmol/g were classified as severe iron overload according to Liver-Iron MR protocol of Gandon et al. [18]. The Liver and Iron–MR protocol and calculation algorithm are available at http://www.radio.univ-rennes1.fr.

Pre-transplant MRI measurements of SIC, BIC and PIC were calculated from the same MRI sequences used for pre-transplant LIC. The images were kept in our archives, unmodified for future consultations. Pre-transplant MRI was performed on average 18 days before transplant. The first, second, and third post-transplant MRI measurements of LIC, SIC, BIC and PIC were obtained at 1–6, 6–12 and 12–24 months, respectively. Patients who underwent HSCT were divided into two groups, those with pre-transplant LIC < 100 µmol/g (Group 1) and LIC > 100 µmol/g (Group 2).

All patients with moderate or severe iron overload had undergone intravenous chelation therapy with deferoxamine (30 mg/kg/day in continuous infusion) from admission until discharge from the Transplant Center. Outpatients had continued chelation oral therapy (deferasirox, 15–20 mg/kg/day, dose adjusted in case of adverse effects). We don’t use phlebotomy routinely, because poorly tolerated by little patients.

The control group consisted of 20 pediatric patients (10 girls and 10 boys, age 1–17 years, mean age 10.75 ± 4.8 years), with no history of any acute or chronic disease or blood transfusion, who underwent MRI for orthopedic diseases, traumas, or as follow-up after leg correction surgery. LIC, SIC, BIC and PIC in this group were measured by MRI to establish normal values.

Clinical data were obtained from the medical records of all transplanted patients. Primary endpoints were overall survival, transplant-related mortality, disease recurrence, quality of life (Karnofsky-Lansky scale) and time to engraftment. Secondary endpoints were early complications (SOS, acute GVHD, infections) and long-term effects (chronic GVHD, osteoporosis, pancreatic insufficiency, chronic infections, secondary graft failure).

### Statistical analysis

Continuous variables were expressed as mean ± standard deviation (SD) or mean ± standard error of the mean (SEM), and categorical variables as frequency, absolute value or percentage. Results were compared by non-parametric statistics. Spearman’s rank correlation coefficient was used to assess the relationship between MR and clinical parameters, the Mann-Whitney *U*-test was used to compare unpaired data in groups of patients, and the Friedman test was used for repeated measures analysis of variance to detect differences in iron overload during bone marrow transplantation. Multiple regression analysis was performed to assess the relationships among multiple MR-independent variables and clinical outcomes. Fisher’s exact test was performed to assess the strength of association between categorical variables, with results reported as odds ratios, sensitivity and specificity.

All statistical tests were two-sided, with *p* values < 0.05 considered statistically significant. Statistical analysis was performed using WinStat software (v.2012.1, R. Fitch).
